# Supplementary material for: A Plant Biostimulant from Ascophyllum nodosum Potentiates Plant Growth Promotion and Stress Protection Activity of Pseudomonas protegens CHA0
Source: Plants (Basel). 2023 Mar 7;12(6):1208. doi: 10.3390/plants12061208 (PMC10053968; doi:10.3390/plants12061208)
Supplement: Supplementary file 1 [file plants-12-01208-s001.zip › plants-2165975-supplementary.pdf]

Table S1. Media used for growing the different bacterial cultures.

No.	Bacterial Species	Growth media
1	*Azospirillum lipoferum* 1842	Nutrient agar (NA)
2	*Azotobacter vinelandii* (ATCC12837)	Potato dextrose agar (PDA)
3	*Bacillus subtilis*	Yeast extract mannitol (YEM) agar
4	*Bacillus thuringiensis* subsp. *oloke*	Mannitol egg yolk polymyxin (MEYP) agar
5	*Bradyrhizobium japonicum* 3I1b6	Nutrient agar (NA)
6	*Enterobacter agglomerans* (ATCC23216)	Nutrient agar (NA)
7	*Enterobacter cloacae* CAL2	Nutrient agar (NA)
8	*Kluyvera ascorbata* SUD165	MRS (deMan, Rogosa, and Sharpe) agar
9	*Lactobacillus acidophilus* (ATCC 4356)	Yeast mannitol agar (YMA)
10	*Paenibacillus polymyxa* K56	Tryptic soy agar (TSA)
11	*Pseudomonas brassicacearum* ZY-2-1	Tryptic soy agar (TSA)
12	*Pseudomonas fluorescens* 34-13	Luria-Bertani (LB) agar
13	*Pseudomonas protegens* CHAO	Tryptic soy agar (TSA)
14	*Pseudomonas putida* (ATCC 12633)	Yeast mannitol agar (YMA)
15	*Sinorhizobium fredii* (ATCC51808)	Luria-Bertani (LB) agar
16	*Sinorhizobium meliloti* RM11559	Mitis Salivarius Agar with Tellurite (MSAT)
17	*Streptococcus salivarius* C699	Nutrient agar (NA)

Table S2. List of primers used in the qPCR experiment.

**Name of the gene*; gene ID**	Forward primers	Reverse primers
hcnA; 57475633	5′- CCGTCCTCAGCGTGATCC-3′	5′- CGGCTTGACCAGGGTCTG-3′
WspR; 57474139	5′- GCCAACGACTACCTGGTCAAG-3′	5′- GTCGGAGTTCATCAGCCGTTG -3′
cheW; 57474134	5′- AAGCAAGTGGTCGGGATCAT -3′	5′- GTTCCGACCATTCCTCTTCG -3′
pvdS; 57477257	5′- AGACGTGGTTCAAGATGCGT -3′	5′- TCAGGTGAAGCGCCATGAAT -3′
rpoC; 57478537	5′- ACCCAGGGCGAGAAGTACAA- 3′	5′- CCCGAGTCAGCCATCATGTA- 3′

**hcnA*, cyanide-forming glycine dehydrogenase subunit HcnA; *WspR*, signal transduction system regulator diguanylate cyclase; *cheW*, chemotaxis protein; *pvdS* RNA polymerase sigma-70 factor, ECF subfamily; *rpoC*, DNA-directed RNA polymerase subunit beta.
